# A Pediatric Case of Basidiobolomycosis Presenting With an Abdominal Mass

**DOI:** 10.7759/cureus.25986

**Published:** 2022-06-16

**Authors:** Hossam E Shaaban, Mohammed A Almatrafi, Abdulwahab Telmesani

**Affiliations:** 1 Gastroenterology, National Hepatology and Tropical Medicine Research Institute, Cairo, EGY; 2 Department of Pediatrics, Umm Al-Qura University, Makkah, SAU; 3 Pediatric Gastroenterology, Security Forces Hospital - Makkah, Makkah, SAU

**Keywords:** abdominal mass, voriconazole, itraconazole, basidiobolus ranarum, basidiobolomycosis

## Abstract

Basidiobolomycosis is a rare fungal infection caused by *Basidiobolus ranarum. *The condition has been reported in children and adults presenting with abdominal pain, weight loss, abdominal distension, vomiting, diarrhea, fever, and an abdominal mass. We report a case of a previously healthy 2.5 years old male who presented to the ER complaining of abdominal pain and distension for two weeks together with significant weight loss. He looked ill and cachectic. He had tachycardia but was afebrile. His abdominal examination showed a right-sided abdominal mass. His initial abdominal ultrasound (US) suggested an abdominal tumor. The patient was referred to a tertiary center where he had an ultrasound-guided biopsy that showed fungal hyphae consistent with basidiobolomycosis.

## Introduction

Basidiobolomycosis is a rare fungal infection caused by *Basidiobolus ranarum*, an environmental saprophyte found worldwide, common in soil, decaying vegetable matter, and dungs of amphibians, reptiles including geckos, fish, and insectivorous bats [1-3]. *Basidiobolus haptosporus*-like fungus has been described as a potential causative agent for gastrointestinal basidiobolomycosis in a recent report [4]. The condition has been reported in children and adults, infecting primarily immunocompetent hosts. It is extremely rare in the immunocompromised hosts contrary to the cases with mucormycosis who are almost always immunosuppressed. There are no prominent risk factors for basidiobolomycosis infection. However, most of the cases were found in warm rural and tropical areas, which are optimal for fungal growth that needs a hot and humid atmosphere. Many animals implicated in fungal transmission also live in those areas [5,6]. The primary sites of infection are subcutaneous tissues and the gastrointestinal tract [7]. The presumed portal of entry for gastrointestinal infection is ingestion of soil, animal feces, and food contaminated by either, as well as rectal inoculation [8]. The most common presenting symptom is abdominal pain (86.3%), followed by weight loss (33.3%), abdominal distension (16.7%), vomiting (15.7%), and diarrhea (13.7%), with fever reported in 40.2% of patients, lower gastrointestinal bleeding in 14%, and a palpable abdominal mass in 30.4% of cases [9,10]. The laboratory findings in patients with gastrointestinal bleeding commonly include neutrophilic leucocytosis, eosinophilia, and a high erythrocyte sedimentation rate (ESR) [10]. The most commonly involved organ in gastrointestinal bleeding is the colon (82%), followed by the small bowel (36%). Other less common sites are the stomach, liver, and biliary system [11].

The first recorded human infection caused by *B. ranarum* was in 1956 [12]. Gastrointestinal basidiobolomycosis was diagnosed for the first time in 1980 in a four-year-old child [13], and the first culture proving gastrointestinal basidiobolomycosis was reported in 1986 in the United States. A total of 122 cases were reported worldwide as of 2018, with 76 reported pediatric cases. The reported pediatric cases were mostly from rural areas of tropical countries in Asia, Africa, Latin America, and Arizona in the United States [14]. In fact, there have been many cases reported in the Middle East, very common in certain parts of Saudi Arabia from the adult and pediatric populations mostly from the Southern Western region. This fact should be highlighted to increase the awareness of clinicians in the Middle East including Saudi Arabia [8,14,15].

## Case presentation

A previously healthy two-and-a-half-year-old male presented to the ER complaining of abdominal pain, abdominal distension, and weight loss for the previous two weeks. The disease started with a documented fever of 38°C (at home) and vomiting for a day with decreased oral intake and activity. The abdominal pain and distension started immediately afterward. The pain was generalized all over the abdomen. The pain started suddenly, was steady, and woke him up. He had abdominal distention and constipation, which was only relieved by enema or glycerin suppositories. The patient suffered significant weight loss from 15 kg to 9.5 kg and a decrease in activity through the disease course with a loss of appetite to the extent that he could only tolerate milk or feeding by syringe. His past medical and surgical history was unremarkable. His family history was irrelevant. Upon physical examination, the patient was conscious, cachectic, and pale and looked ill. His vital signs upon admission were as follows: a pulse rate of 109 beats per minute, blood pressure of 105/66 mmHg, respiratory rate of 25 per minute, and temperature of 36.8°C. Abdominal examination showed an obvious asymmetrical distention on the right side of the abdomen. The umbilicus was everted. When palpated, there was a generalized tenderness over the abdomen, more on the right side, with a palpable mass in the right lumbar region under the costal margin crossing the midline at the umbilicus, which was circumscribed, firm, tender, and dull on percussion. There were no palpable enlarged lymph nodes.

The initial laboratory workup revealed a white blood cell (WBC) count of 19.8 k/uL, 58% neutrophils (NE), hemoglobin (HB) of 10.1 g/dL, platelets (PLT) of 787 k/uL, C-reactive protein (CRP) of 30.41 mg/dL, erythrocyte sedimentation rate (ESR) of 70 mm/hour, and albumin of 2.7 g/dL (Table 1).

**Table 1:** Laboratory results

Component	Unit	Patient's result	Standard range
WBC count	×10^3^/uL	19.82	4-12
RBC count	×10^6^/uL	4.61	4-5.3
Hemoglobin	g/dL	8.1	11.5-14.5
Hematocrit	%	26	33-43
MCV	fL	56.4	76-90
MCH	pg	17.6	25-31
MCHC	g/dL	31.2	29-31
RDW	%	15.6	11.5-15
Platelet count	×10^3^/uL	787	150-490
MPV	fL	8.4	7.4-10.4
Neutrophils #	×10^3^/uL	10.56	1.5-8
Neutrophils	%	53.3	37-80
Lymphocytes #	×10^3^/uL	3.43	1.9-9.8
Lymphocytes	%	17.3	10-50
Monocytes #	×10^3^/uL	1.47	0-0.9
Monocytes	%	7.4	0-12
Eosinophils #	×10^3^/uL	4.3	0.1-1
Eosinophils	%	21.7	0.6-7.3
Basophils #	×10^3^/uL	0.06	0-0.1
Basophils	%	0.3	0-1.7
Prothrombin time	Seconds	15.3	11-15
INR		1.17	0.89-1.1
AST	U/L	26	5-34
ALT	U/L	18.7	0-55
ALP	U/L	110	<500
Bilirubin total	mg/dL	0.24	0.2-1.2
Bilirubin (direct)	mg/dL	0.13	<0.5
Protein	g/dL	6.47	5.6-7.5
Albumin	g/dL	2.7	3.8-5.4
GGT	U/L	42	12-64
Creatinine	mg/dL	0.46	0.3-0.7
Urea	mg/dL	9	11-36
Sodium	mmol/L	131	136-145
Potassium	mmol/L	3.61	3.4-4.7
Erythrocyte sedimentation rate	mm/hour	70	≤10
C-reactive protein	mg/dL	30.41	0-0.5

**Figure 1:**
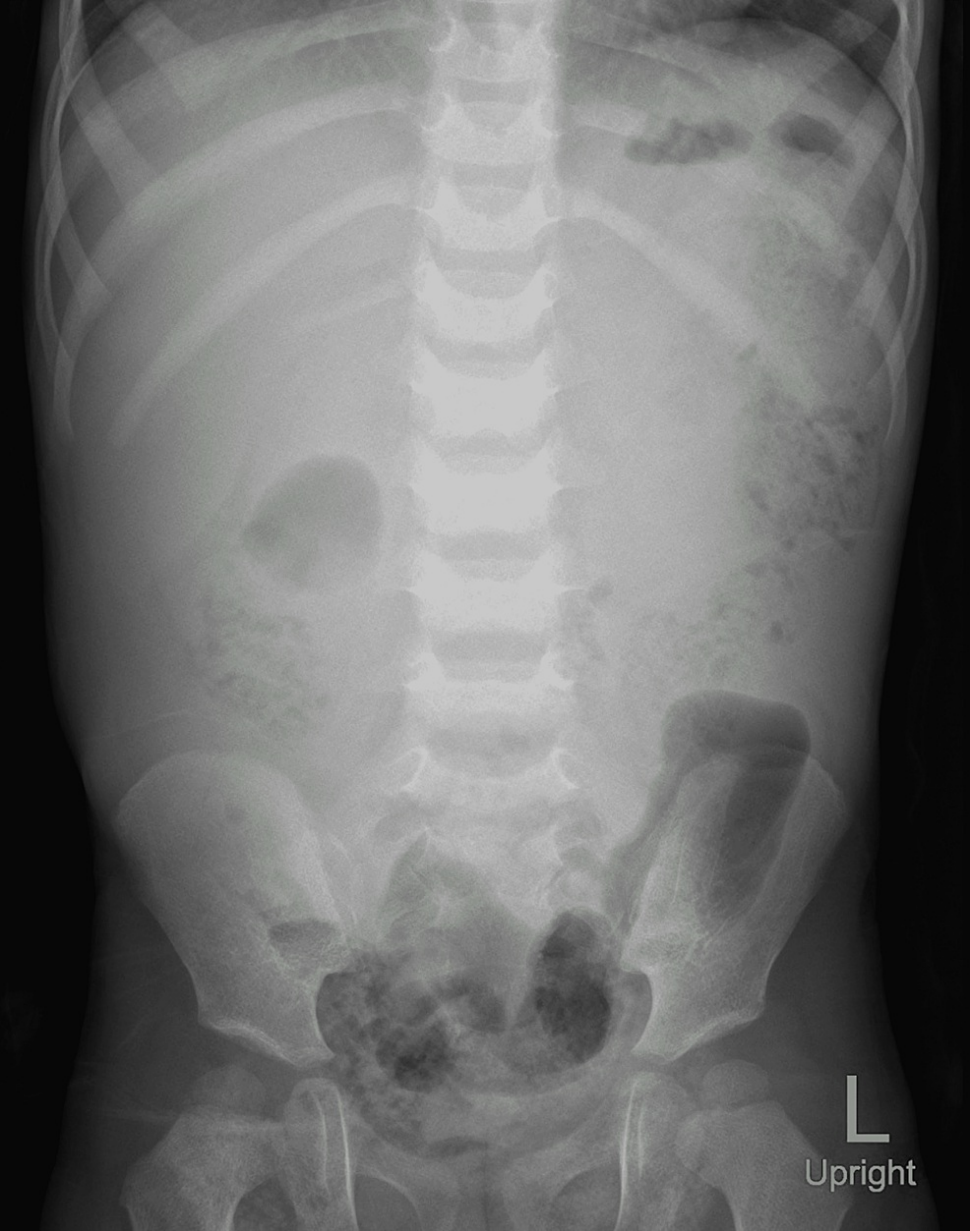
Digital X-ray of the abdomen in upright position revealing no evidence of air/fluid level

An abdominal X-ray showed no air/fluid levels and no air under the diaphragm (Figure 1).

**Figure 2:**
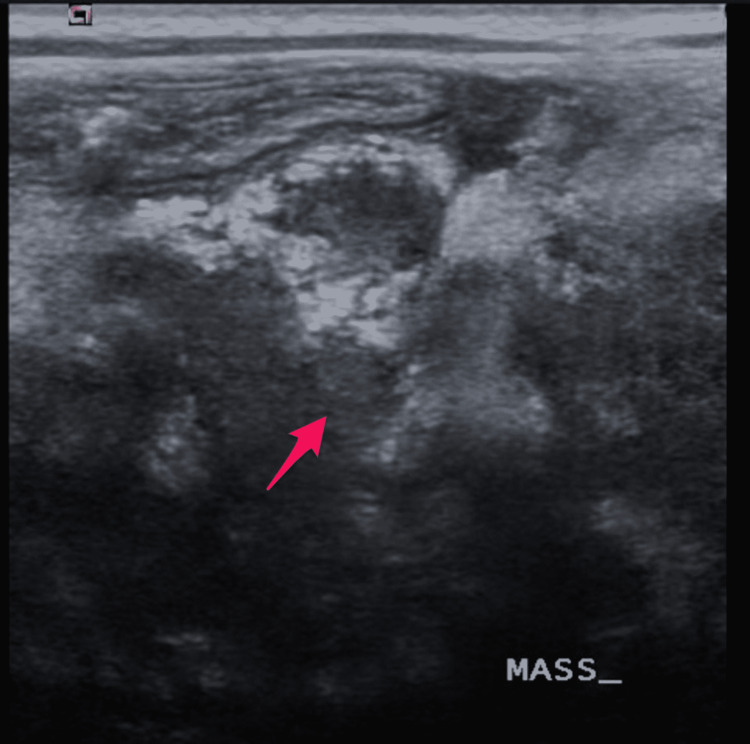
Grayscale US of the abdomen using a superficial probe (7.5 Hs) revealing large pelvic-abdominal heterogeneously solid mass lesion measuring about 10.8 × 6.8 × 7 cm along its maximum CC, TS, and AP diameters, respectively (arrow)

The abdominal ultrasound (US) was suggestive of a tumor (Figure 2).

**Figure 3:**
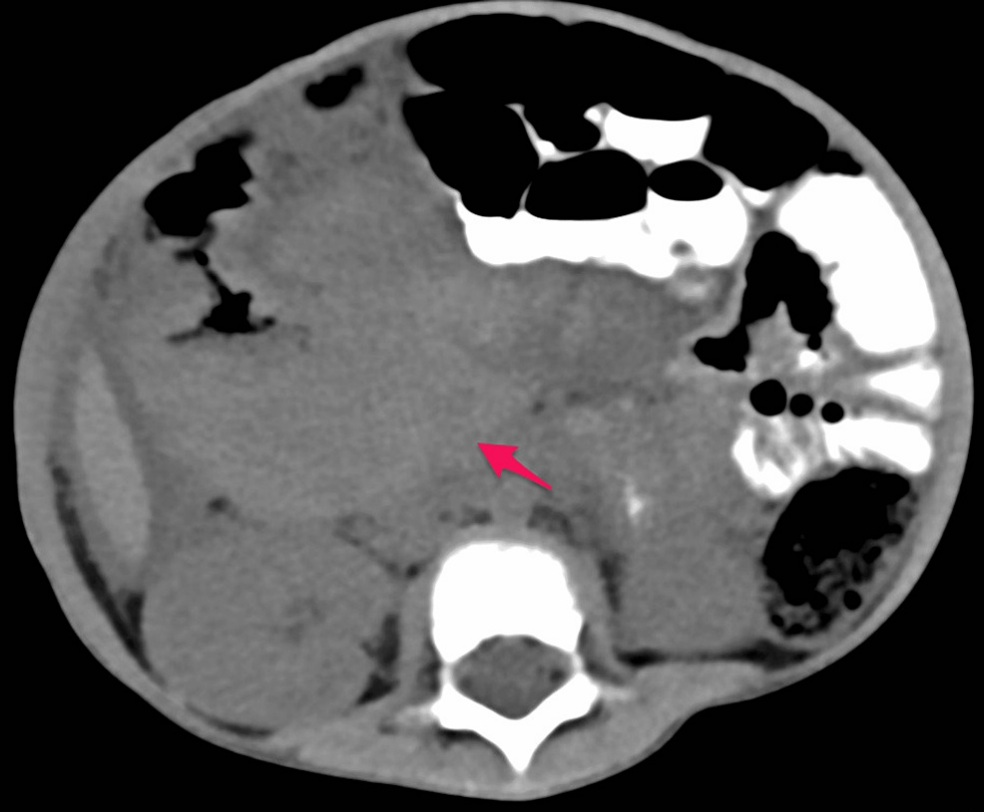
Pre-contrast phase showing a heterogeneous density with no calcification (arrow)

**Figure 4:**
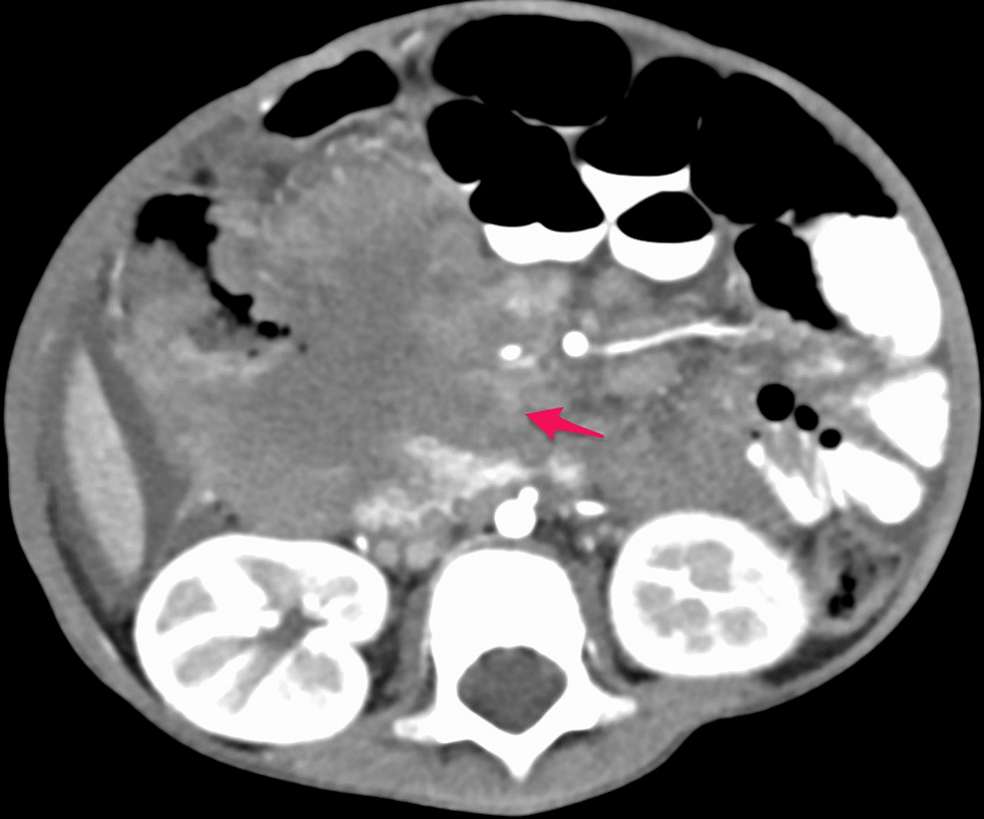
Post-contrast axial image in arterial phase showing heterogeneous enhancement of the mass (arrow)

**Figure 5:**
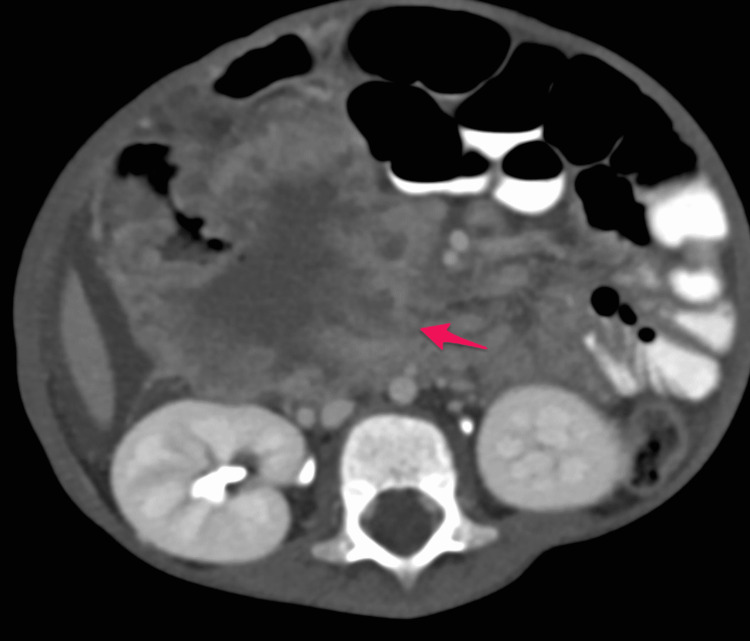
Post-contrast axial image in venous phase showing heterogeneous enhancement of the mass (arrow)

**Figure 6:**
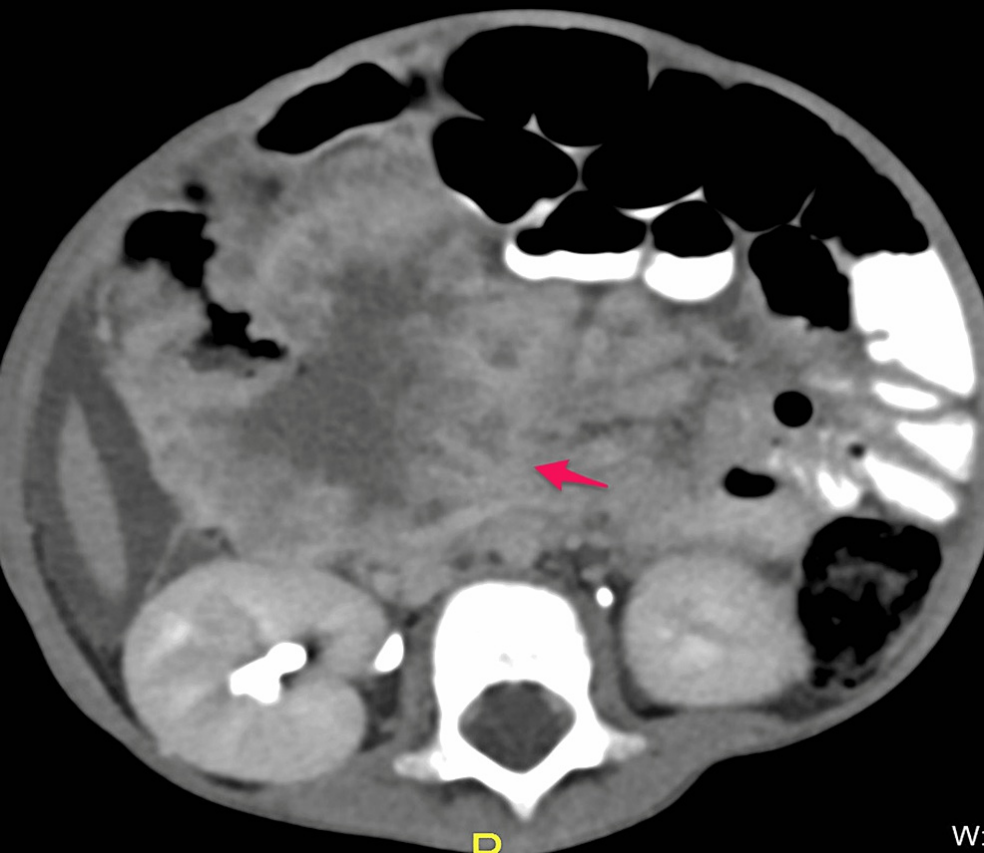
Post-contrast axial image in delayed phase showing increased enhancement of the mass (arrow)

**Figure 7:**
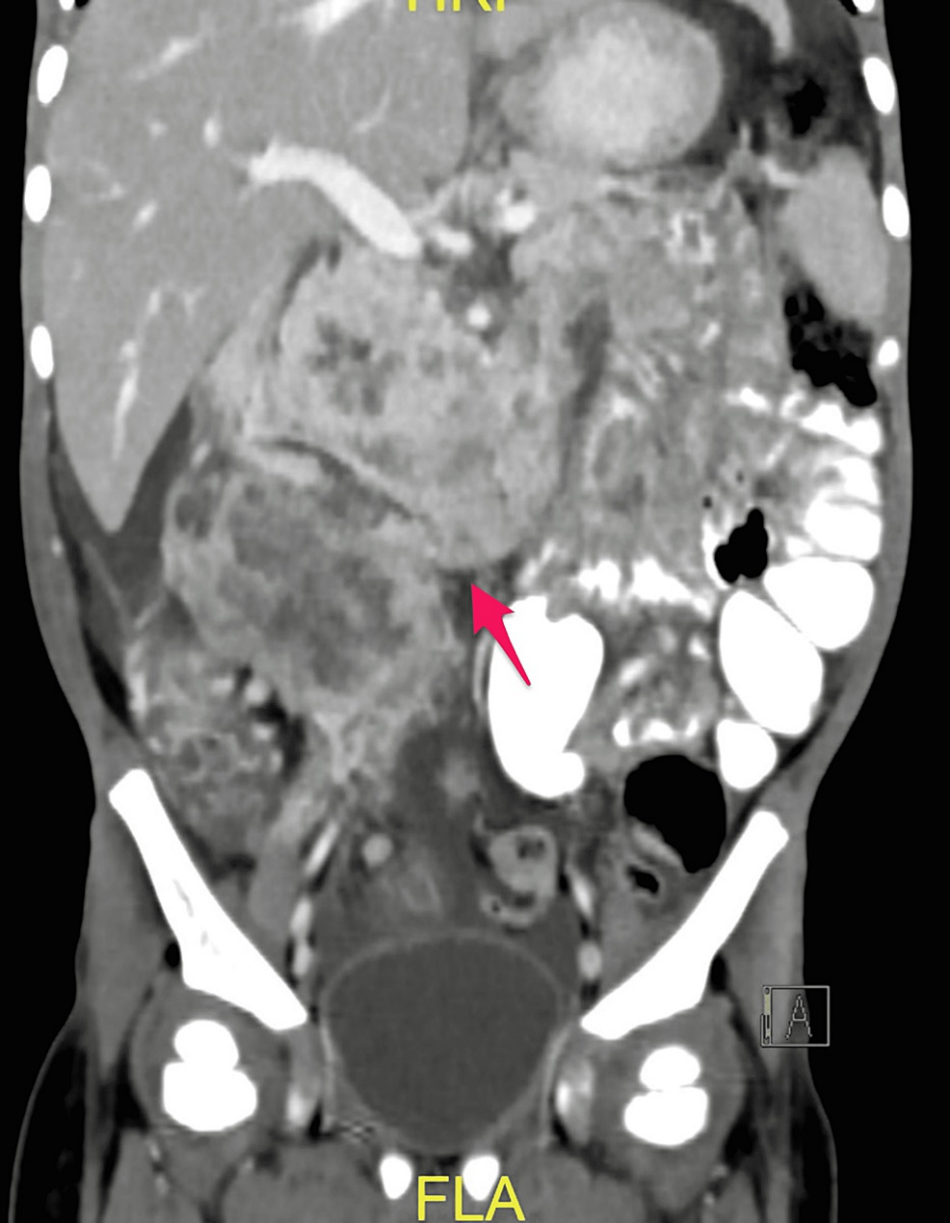
Post-contrast coronal reformatted image in venous phase showing the mass involving the ascending colon, which shows marked enhancing mural thickening and hepatic flexure (arrow)

The patient was initially admitted to the ward. He received an IV antibiotic (cefotaxime 50 mg/kg q8 hours) as well as IV D5NS. He underwent an abdominal CT with oral and intravenous contrast in a triphasic manner, which showed a large well-defined soft tissue mass measuring about (8.5 × 7.5 cm) and occupying most of the right hypochondriac and right iliac regions (Figures 3-7).

The initial differential diagnosis at our hospital was intra-abdominal neoplasia, such as neuroblastoma, Wilms' tumor, rhabdomyosarcoma, and lymphoma. We did not do a PPD test as the provisional diagnosis was a neoplastic lesion rather than abdominal tuberculosis. The patient was referred to a tertiary center for further investigation, and workup at the interventional radiology service was unavailable at that time at our hospital. The patient underwent a US-guided biopsy from the abdominal mass. The result showed granulomatous inflammation with fungal hyphae consistent with basidiobolomycosis. Unfortunately, further data about the patient's treatment course and outcome in the tertiary center could not be obtained.

## Discussion

Colonic involvement in gastrointestinal basidiobolomycosis is commonly misdiagnosed as malignant mass commonly in the cecal or ascending colon with local invasion and, at times, involvement of distant sites [8,16]. Disseminated gastrointestinal basidiobolomycosis is rare but potentially fatal [17]. The condition is most commonly diagnosed post-surgery and after the analysis of the resected tissues. So, the final diagnosis frequently comes late in the disease course [8,16]. As a result, gastrointestinal basidiobolomycosis should be kept in the differential diagnosis of inflammatory diseases of the gastrointestinal tract. In our case, the patient was initially suspected in our hospital to have intra-abdominal neoplasia, such as neuroblastoma, Wilms' tumor, rhabdomyosarcoma, and lymphoma. A definitive diagnosis was obtained by a US-guided biopsy of the mass.

The definitive diagnosis is a fungal culture obtained from the infected tissue, but histopathology can diagnose the condition in many cases. PCR is a new accurate method [18]. For our case, the diagnosis was made through histological examination, which showed typical branching fungal hyphae. Fungal culture was required for the identification of the fungal species.

The optimal treatment is a combined medical and surgical one. All the affected bowel segments should be surgically resected, followed by antifungal treatment for at least three months [3]. Itraconazole is the antifungal treatment of choice. Recently, voriconazole has been successfully used [19].

## Conclusions

Gastrointestinal basidiobolomycosis is a rare, difficultly diagnosed disease due to its nonspecific presentation that often masquerades as invasive malignancy. Increased awareness among physicians is needed for proper diagnosis and treatment.

## References

[1] Kombaté K, Saka B, Mouhari-Toure A (2012). [Basidiobolomycosis: a review]. Med Sante Trop.

[2] Paré JA (2003). Fungal diseases of amphibians: an overview. Vet Clin North Am Exot Anim Pract.

[3] Saadah OI, Farouq MF, Daajani NA, Kamal JS, Ghanem AT (2012). Gastrointestinal basidiobolomycosis in a child; an unusual fungal infection mimicking fistulising Crohn's disease. J Crohns Colitis.

[4] Bshabshe AA, Joseph MR, Hakami AM, Azraqi TA, Humayed SA, Hamid ME (2020). Basidiobolus haptosporus-like fungus as a causal agent of gastrointestinal basidiobolomycosis. Med Mycol.

[5] Atadokpédé F, Gnossikè J, Adégbidi H (2017). Cutaneous basidiobolomycosis: seven cases in southern Benin. Ann Dermatol Venereol.

[6] Geramizadeh B, Heidari M, Shekarkhar G (2015). Gastrointestinal basidiobolomycosis, a rare and under-diagnosed fungal infection in immunocompetent hosts: a review article. Iran J Med Sci.

[7] Mohta A, Neogi S, Das S (2011). Gastrointestinal mucormycosis in an infant. Indian J Pathol Microbiol.

[8] Nemenqani D, Yaqoob N, Khoja H, Al Saif O, Amra NK, Amr SS (2009). Gastrointestinal basidiobolomycosis: an unusual fungal infection mimicking colon cancer. Arch Pathol Lab Med.

[9] Pezzani MD, Di Cristo V, Parravicini C (2019). Gastrointestinal basidiobolomycosis: an emerging mycosis difficult to diagnose but curable. Case report and review of the literature. Travel Med Infect Dis.

[10] Vikram HR, Smilack JD, Leighton JA, Crowell MD, De Petris G (2012). Emergence of gastrointestinal basidiobolomycosis in the United States, with a review of worldwide cases. Clin Infect Dis.

[11] Al Qahtani S, Alangari A, Mohammed N, Albarrag A, Elzein F (2019). Colonic basidiobolomycosis presenting with intestinal obstruction and a normal eosinophil count. IDCases.

[12] Kian Joe L, Pohan A, Tjoei Eng NI, van der Meulen H (1956). Basidiobolus ranarum as a cause of subcutaneous mycosis in Indonesia. AMA Arch Derm.

[13] Hussein MR, Musalam AO, Assiry MH, Eid RA, El Motawa AM, Gamel AM (2007). Histological and ultrastructural features of gastrointestinal basidiobolomycosis. Mycol Res.

[14] Mohammadi R, Ansari Chaharsoghi M, Khorvash F (2019). An unusual case of gastrointestinal basidiobolomycosis mimicking colon cancer; literature and review. J Mycol Med.

[15] Arabi RI, Aljudaibi A, Shafei BA, AlKholi HM, Salem ME, Eibani KA (2019). Paediatric case of gastrointestinal basidiobolomycosis mimicking appendicitis - case report. Int J Surg Case Rep.

[16] Balkhair A, Al Wahaibi A, Al-Qadhi H, Al-Harthy A, Lakhtakia R, Rasool W, Ibrahim S (2019). Gastrointestinal basidiobolomycosis: beware of the great masquerade a case report. IDCases.

[17] Sharma A, Saxena R, Sinha A, Singh S, Yadav T (2019). Disseminated gastrointestinal basidiobolomycosis (GIB) in an infant from western India. Med Mycol Case Rep.

[18] Gómez-Muñoz MT, Fernández-Barredo S, Martínez-Díaz RA, Pérez-Gracia MT, Ponce-Gordo F (2012). Development of a specific polymerase chain reaction assay for the detection of Basidiobolus. Mycologia.

[19] Albaradi BA, Babiker AM, Al-Qahtani HS (2014). Successful treatment of gastrointestinal basidiobolomycosis with voriconazole without surgical intervention. J Trop Pediatr.

